# Voxel-based statistical analysis and quantification of amyloid PET in the Japanese Alzheimer’s disease neuroimaging initiative (J-ADNI) multi-center study

**DOI:** 10.1186/s13550-019-0561-2

**Published:** 2019-09-18

**Authors:** Go Akamatsu, Yasuhiko Ikari, Akihito Ohnishi, Keiichi Matsumoto, Hiroyuki Nishida, Yasuji Yamamoto, Michio Senda, Japanese Alzheimer’s Disease Neuroimaging Initiative

**Affiliations:** 1 Division of Molecular Imaging, Institute of Biomedical Research and Innovation (IBRI), Kobe, Japan; 2 Division of Molecular Imaging, Kobe City Medical Center General Hospital, Kobe, Japan; 3 National Institute of Radiological Sciences (NIRS), National Institutes for Quantum and Radiological Science and Technology (QST), Chiba, Japan; 4 Department of Radiology, Kakogawa Central City Hospital, Kakogawa, Japan; 5 Department of Radiological Technology, Faculty of Medical Science, Kyoto College of Medical Science, Kyoto, Japan; 6 Department of Biosignal Pathophysiology, Graduate School of Medicine, Kobe University, Kobe, Japan; 7 Medical Center for Student Health, Kobe University, Kobe, Japan

**Keywords:** PET, Amyloid, ^11^C-PiB, Voxel-based statistical analysis, *Z*-score

## Abstract

**Background:**

Amyloid PET plays a vital role in detecting the accumulation of in vivo amyloid-β (Aβ). The quantification of Aβ accumulation has been widely performed using the region of interest (ROI)-based mean cortical standardized uptake value ratio (mcSUVR). However, voxel-based statistical analysis has not been well studied. The purpose of this study was to examine the feasibility of analyzing amyloid PET scans by voxel-based statistical analysis. The results were then compared to those with the ROI-based mcSUVR. In total, 166 subjects who underwent ^11^C-PiB PET in the J-ADNI multi-center study were analyzed. Additionally, 18 Aβ-negative images were collected from other studies to form a normal database. The PET images were spatially normalized to the standard space using an adaptive template method without MRI. The mcSUVR was measured using a pre-defined ROI. Voxel-wise *Z*-scores within the ROI were calculated using the normal database, after which *Z*-score maps were generated. A receiver operating characteristic (ROC) analysis was performed to evaluate whether *Z*-sum (sum of the *Z*-score) and mcSUVR could be used to classify the scans into positive and negative using the central visual read as the reference standard. PET scans that were equivocal were regarded as positive.

**Results:**

Sensitivity and specificity were respectively 90.8% and 100% by *Z*-sum and 91.8% and 98.5% by mcSUVR. Most of the equivocal scans were subsequently classified by both *Z*-sum and mcSUVR as false negatives. *Z*-score maps correctly delineated abnormal Aβ accumulation over the same regions as the visual read.

**Conclusions:**

We examined the usefulness of voxel-based statistical analysis for amyloid PET. This method provides objective *Z*-score maps and *Z*-sum values, which were observed to be helpful as an adjunct to visual interpretation especially for cases with mild or limited Aβ accumulation. This approach could improve the Aβ detection sensitivity, reduce inter-reader variability, and allow for detailed monitoring of Aβ deposition.

**Trial registration:**

The number of the J-ADNI study is UMIN000001374

## Background

Although accumulation of amyloid-β (Aβ) plaque in the cerebral cortex is not specific to Alzheimer’s disease (AD), it is believed to begin more than 10 years before the onset of cognitive impairment [1]. Amyloid PET imaging plays a vital role in the detection of in vivo Aβ accumulation as an imaging biomarker of AD [2, 3]. For amyloid PET, visual interpretation is the standard clinical practice to classify Aβ-positive or Aβ-negative in accordance with a neuropathological diagnosis [4–6]. Meanwhile, quantitative analysis of amyloid PET has been widely performed in conjunction with visual interpretation. Quantitative approaches may provide additional diagnostic information, increase diagnostic confidence of any visual interpretation [7], reduce inter-reader variability [8], and provide a longitudinal assessment of Aβ deposition in anti-Aβ therapeutic clinical trials [9] even though such approaches are still limited to research purposes [4, 5].

There are several methods to quantify Aβ accumulation using amyloid PET [10–12]. A commonly used approach is the region of interest (ROI)-based analysis with standardized uptake value ratio (SUVR) between the target regions and a reference region. The cerebellar cortex, whole cerebellum, pons, and cerebral white matter have been used as reference regions as they are considered free of any abnormal fibrillar Aβ deposition [13, 14]. The ROI-based approach has been the de facto standard of quantitative analysis for amyloid PET because of its simplicity.

On the other hand, a voxel-based approach can also be used to analyze amyloid PET. Kemppainen et al. and Ziolko et al. both used statistical parametric mapping (SPM) software to visualize the difference in Aβ accumulation between AD patients and healthy control subjects [15, 16]. However, both these studies involved a limited number of subjects (total number of subjects < 30) and did not show individual maps, looking only at differences between the groups. To our knowledge, there has been no large-scale study conducted that examines the feasibility of voxel-based statistical analysis in assessing amyloid PET.

When looking to apply the voxel-based statistical analysis to amyloid PET, there were two main issues we needed to overcome. The first issue was that we needed to acquire a large dataset that contained both amyloid PET and high-resolution MRI to create a reliable normal database. This issue was resolved by obtaining a large dataset from large-scale multi-center studies on dementia, such as ADNI and J-ADNI [17–19]. These studies provide us with an open database of amyloid PET and high-resolution MRI for a large number of AD patients, mild cognitive impairment (MCI) subjects, and normal control subjects.

The second issue was the spatial normalization of amyloid PET. This was challenging if the subject data lacked MRI, and only PET was available as uptake patterns between Aβ-positive and Aβ-negative images are strikingly different. Although high-resolution MRI has been utilized for spatial normalization of amyloid PET [20], PET-only spatial normalization would be better in view of widespread applications in clinical practice. The spatial normalization issue of amyloid PET was solved using an adaptive template approach [21–24]. The adaptive template method provides us with robust and accurate spatial normalization of amyloid PET without MRI.

These effective breakthroughs have made it possible to create a reliable normal database and then to verify the feasibility of voxel-based analysis of amyloid PET. In this study, we investigated the feasibility of voxel-based statistical analysis of amyloid PET. We then compared the results with ROI-based quantitative analysis.

## Methods

### Subjects

We examined a total of 166 subjects [46 mild AD patients; 62 mild cognitive impairment (MCI) subjects; 58 normal control (NC) subjects] who underwent ^11^C-PiB PET scans. These data were obtained from the Japanese Alzheimer’s Disease Neuroimaging Initiative (J-ADNI) database, which are available from the National Bioscience Database Center Human Database, Japan (Research ID: hum0043.v1, 2016). The J-ADNI was launched in 2007 as a public-private partnership, led by principal investigator Takeshi Iwatsubo, MD. The primary goal of J-ADNI was to test whether serial MRI, PET, other biological markers, and clinical and neuropsychological assessments can be combined to measure the progression of late MCI and mild AD in the Japanese population. The clinical inclusion criteria of neuropsychological tests are described in the article by Iwatsubo et al. [19]. This study was approved by the ethics committee of participating centers, and all subjects had signed an informed consent form for the retrospective data analysis of this kind. The present analysis was also approved by the ethics committee of IBRI, which downloaded the J-ADNI data.

### PET scanners

Table 1 shows PET scanners and the reconstruction parameters used in the J-ADNI study. All PET sites, including their scanners, imaging protocols, and ^11^C-PiB production, were certified by the J-ADNI PET QC core before scanning the first subject. Inter-scanner variability was minimized with the Hoffman three-dimensional (3D) phantom data by optimizing the reconstruction parameters so that the image quality and resolution was assured for each scanner [25].

**Table 1:** PET scanners and reconstruction parameters used in the J-ADNI study

PET scanner		Reconstruction parameters		
Vender	Model	Algorithm	Iteration	Subset
GE	Advance	Iterative (FORE+OSEM)	6	16
GE	Discovery ST Elite Performance	Iterative (VUE Point plus)	4	30
GE	Discovery ST Elite	Iterative (VUE Point plus)	2	40
Philips	GEMINI GXL	LOR RAMLA	2	N/A
Shimadzu	Eminence SOPHIA G/M	FORE+DRAMA	4	N/A
Shimadzu	Eminence SOPHIA G/X	FORE+DRAMA	4	N/A
Shimadzu	Eminence SOPHIA B/L	FORE+DRAMA	4	N/A
Shimadzu	Eminence G/X	FORE+DRAMA	4	N/A
Shimadzu	HEADTOME V	Iterative (FORE+OSEM)	4	16
Siemens	ECAT ACCEL	Iterative (FORE+OSEM)	6	16
Siemens	ECAT EXACT HR47	Iterative (FORE+OSEM)	6	16
Siemens	ECAT EXACT HR+	Iterative (FORE+OSEM)	4	16
Siemens	Biograph 6	Iterative (FORE+OSEM)	4	16
Siemens	Biograph 16	Iterative (FORE+OSEM)	4	14
Siemens	Biograph 16 Truepoint	Iterative (3D)	4	21
Toshiba	Aquiduo	Iterative (FORE+OSEM)	4	14

### Visual interpretation

All ^11^C-PiB PET images were centrally and independently classified into positive, equivocal, and negative by three physicians specializing in nuclear neuroimaging more than 15 years. The visual interpretation criteria are described in the article by Yamane et al. [26]. For those cases where the three physicians’ interpretation did not match, they discussed these in a consensus reading session. A unified read at these sessions was determined as the official interpretation. These visual read results included additional comments by the physicians made during the consensus reading session.

### Generation of the normal database (NDB)

**Fig. 1:**
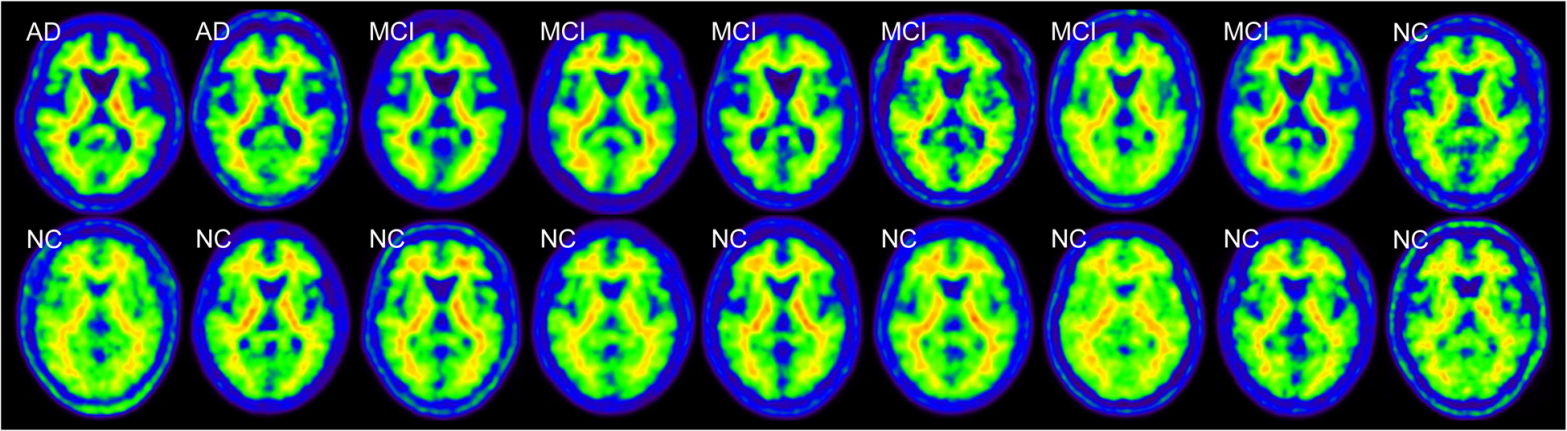
Eighteen cases of Aβ-negative ^11^C-PiB PET images used to create the normal database. The clinical diagnosis of each case is described in the upper left

**Fig. 2:**
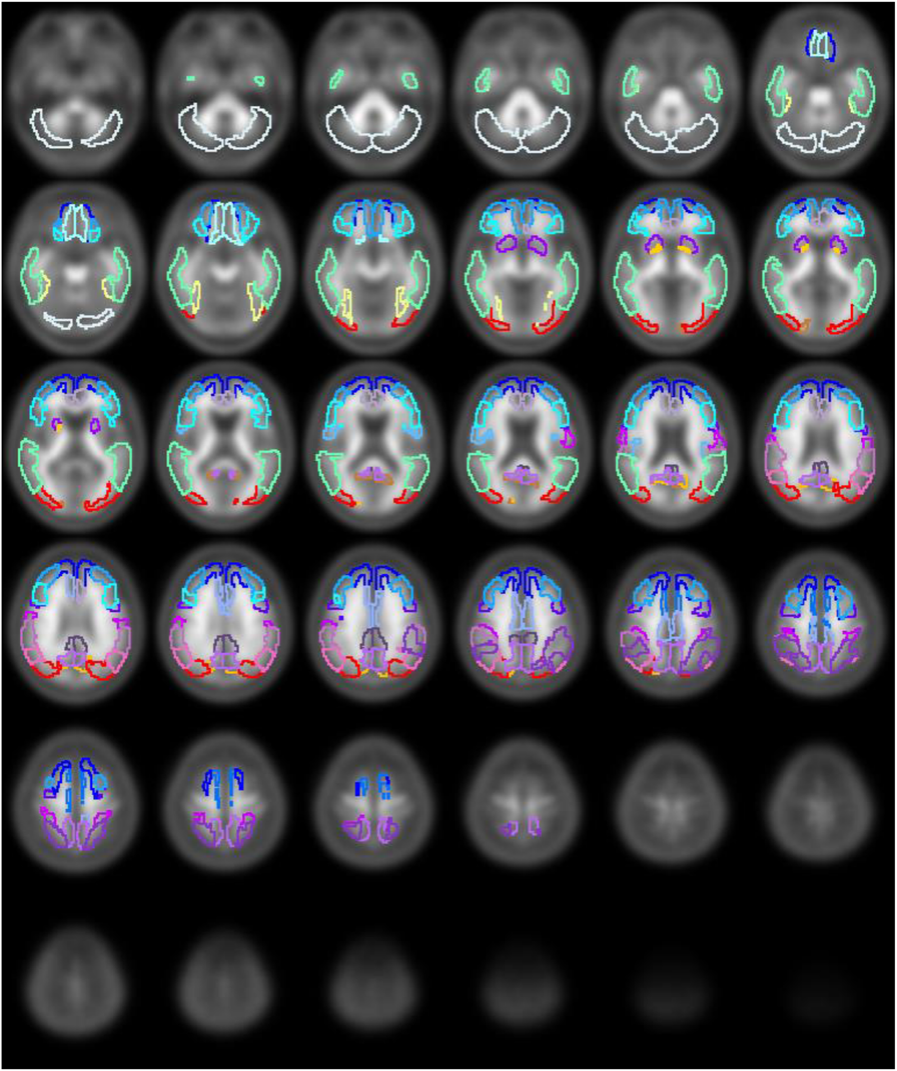
The empirical PiB-prone (EPP) ROI template

**Fig. 3:**
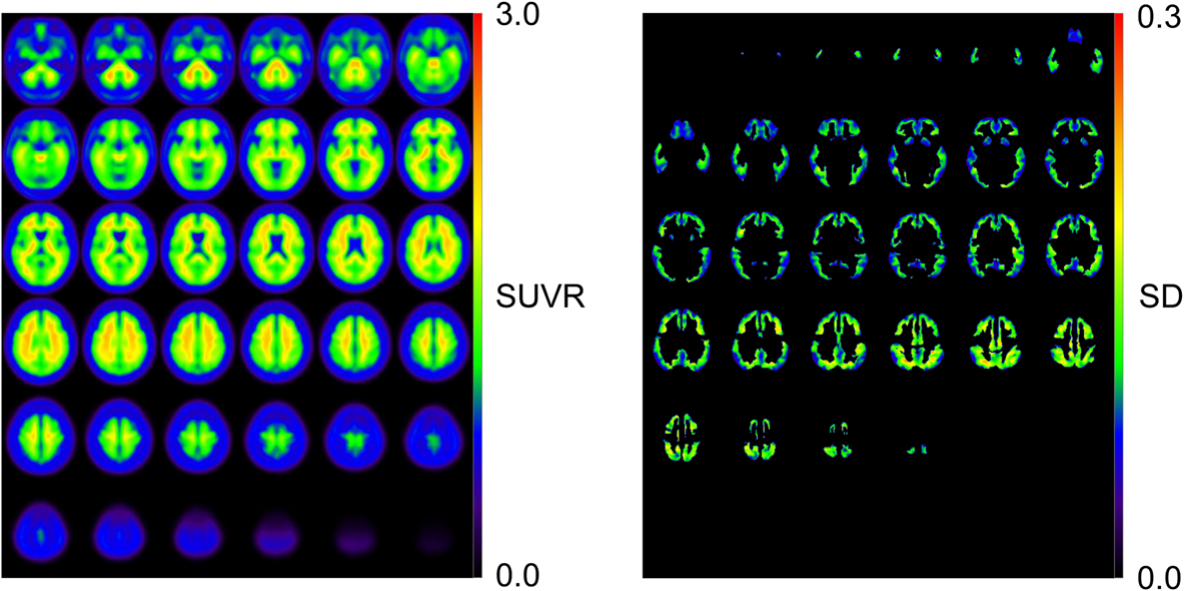
Average (left) and standard deviation (SD) (right) maps created as the normal database. The SD map has been masked by the empirical PiB-prone (EPP) region of interest template

Eighteen Aβ-negative PET images from 2 AD patients, 6 MCI subjects, and 10 NC subjects were used to create the normal database (NDB) of ^11^C-PiB PET (Fig. 1). Table 2 shows mean cortical SUVR (mcSUVR) and maximum SUVR for each ^11^C-PiB PET image, which were calculated by the method described below. The mcSUVRs in all subjects were smaller than 1.45. These ^11^C-PiB PET images were acquired in other studies using the Discovery 690 PET/CT scanner in IBRI [27]. Imaging protocols such as the injection activity (555 MBq), uptake time (50 min), and scan duration (20 min) were the same as those used in the J-ADNI study [28]. The PET images were reconstructed using the 3D ordered subset expectation maximization (OSEM) algorithm (VUE point HD) with 4 iterations and 16 subsets. A Gaussian smoothing filter with 4 mm (*n* = 6) or 5 mm (*n* = 12) full width at half maximum (FWHM) was applied to the PET images. These parameters were determined by the phantom experiment mentioned earlier [29]. We used the PMOD ver. 3.7 and PNEURO tool for image analysis. In order to spatially normalize the PET images, we used the 3D T1-weighted MRI. The PET images were co-registered to the MRI with the normalized mutual information method implemented in the PMOD. The MRI was then spatially normalized to the Montreal Neurological Institute (MNI) T1 template [30, 31]. The co-registered PET images were also spatially normalized to the standard MNI space (matrix dimensions 91 × 109 × 91, voxel size 2 × 2 × 2 mm^3^) using the transformation data of the MRI spatial normalization. After that, the voxel values of the normalized PET images were divided by the mean value of the cerebellar cortex area, which was defined as the reference region in the empirical PiB-prone (EPP) ROI template (Fig. 2) [23]. The EPP-ROI template provided the target region, which characterized voxel-wise amyloid specific regions based on the actual human ^11^C-PiB PET images. Then, average and standard deviation (SD) images were created from the NDB. We picked up some voxels as well the entire EPP-ROI and examined the normality using the Shapiro-Wilk test. As a result, we assumed that the distributions of values per voxel followed the Gaussian distribution. Figure 3 shows the average and SD images after the white matter regions were masked based on the EPP-ROI template.

**Table 2:** Subject characteristics in the normal database

Subjects	Clinical diagnosis	PiB-PET visual classification	mcSUVR	Maximum SUVR in all voxels
1	AD	Negative	1.24	2.36
2	AD	Negative	1.24	2.33
3	MCI	Negative	1.37	2.94
4	MCI	Negative	1.31	2.32
5	MCI	Negative	1.43	2.73
6	MCI	Negative	1.30	2.72
7	MCI	Negative	1.34	2.56
8	MCI	Negative	1.26	2.79
9	NC	Negative	1.29	2.36
10	NC	Negative	1.21	2.38
11	NC	Negative	1.32	2.54
12	NC	Negative	1.35	2.39
13	NC	Negative	1.31	2.68
14	NC	Negative	1.34	2.59
15	NC	Negative	1.29	2.22
16	NC	Negative	1.22	2.21
17	NC	Negative	1.18	2.17
18	NC	Negative	1.26	2.22

### Voxel-based statistical analysis workflow

**Fig. 4:**
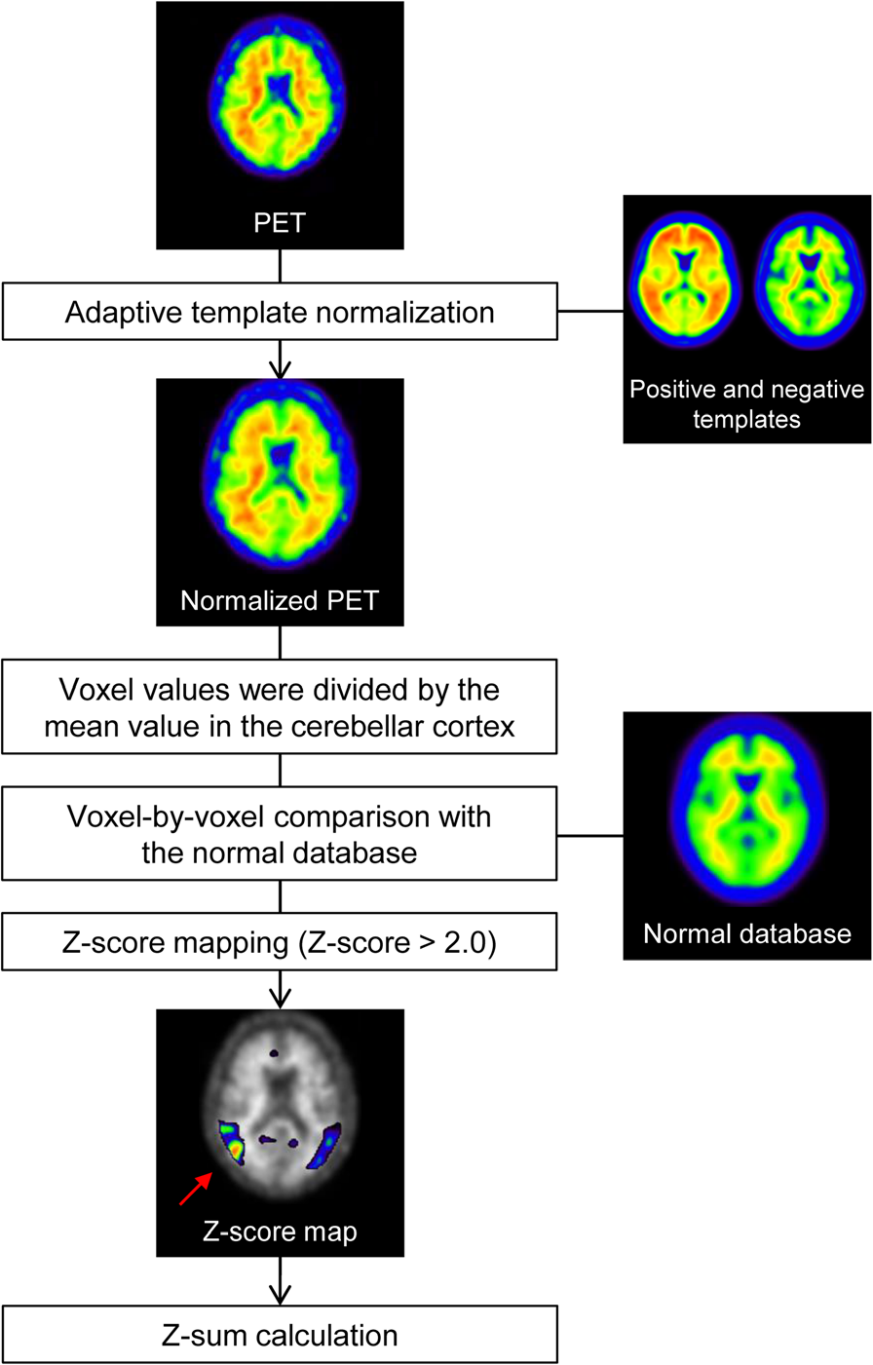
The workflow of the voxel-based statistical analysis

Figure 4 shows the workflow of voxel-based statistical analysis for amyloid PET. Firstly, the PET images were spatially normalized to the MNI space using the PET-only adaptive template normalization method [23]. Then, voxel values were divided by the mean value of the cerebellar cortex area in the same way as the above NDB generation process. After that, the PET images were compared voxel-by-voxel against the NDB, and *Z*-score maps were generated within the EPP-ROI area (Figs. 2 and 3). The *Z*-score was calculated on each voxel using the following equation:

$$ Z-{\mathrm{score}}_i=\frac{x_i-\overline{x}}{\mathrm{SD}} $$

where *x*_*i*_ is the voxel value of the PET image, and $\overline{x}$ and SD are the corresponding voxel value of the average and SD images, respectively, which are derived from the NDB (Fig. 2). After 3D Gaussian smoothing with 8 mm FWHM, *Z*-score values more than 2.0 were superimposed on the gray-scale PET images as a *Z*-score map. In addition to the *Z*-score map, we calculated a sum of the *Z*-score that were more than 2.0 within the EPP-ROI area (*Z*-sum).

### SUVR measurement

In addition to the voxel-based approach, an ROI-based quantitative analysis was also applied to the normalized PET images to compare the results. We calculated a mean cortical standardized uptake value ratio (mcSUVR) within the EPP-ROI template [23]. This ROI template includes the frontal cortex, lateral temporal cortex, parietal cortex, posterior cingulate, precuneus, occipital cortex, and striatum as the target regions and the cerebellar cortex as the reference region. The reference region used was the same as in the voxel-based analysis.

### ROC analysis

A receiver operating characteristics (ROC) analysis was performed to evaluate the capability of *Z*-sum and mcSUVR to classify the scans into positive and negative using the visual read as the reference standard, in which equivocal scans were regarded as positive.

## Results

**Fig. 5:**
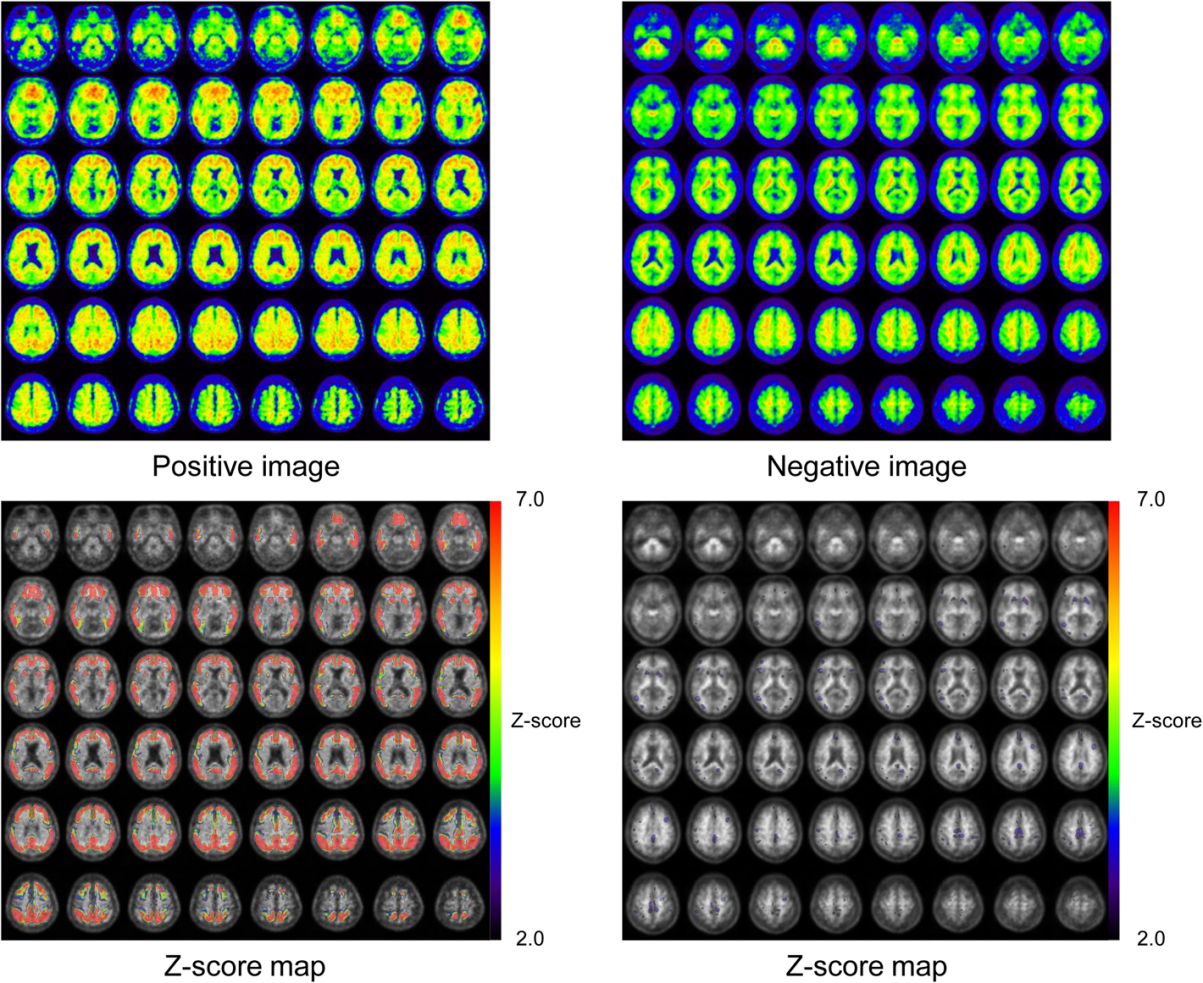
Representative positive and negative cases and their *Z*-score maps depicted in MNI space

**Fig. 6:**
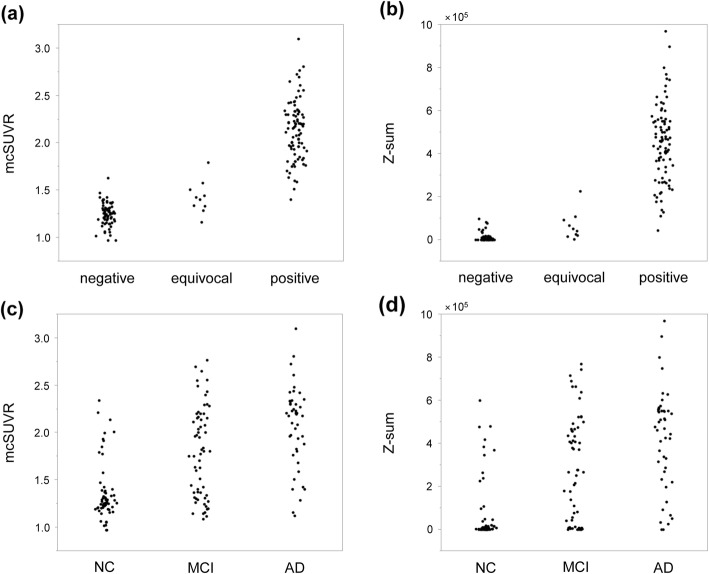
mcSUVR and *Z*-sum values for positive, equivocal, and negative groups (**a**, **b**) as well as those for normal control, mild cognitive impairment, and Alzheimer’s disease groups (**c**, **d**)

The ^11^C-PiB PET images had been visually classified as positive for 88 cases, equivocal for 10 cases, and negative for 68 cases. The proposed voxel-based analysis was successfully used on all PET images. Figure 5 shows the PET images and *Z*-score maps of representative positive and negative cases. The *Z*-scores in most voxels were lower than 2.0 in the negative case, while they were higher than 2.0 in the positive case. Figure 6 shows scatter plots of *Z*-sum and mcSUVR against visual read and clinical diagnosis. The *Z*-sum values for most negative cases were almost zero. ROC analysis was done to assess the differentiation of positive from negative cases. This indicated that sensitivity and specificity were 90.8% and 100% by *Z*-sum with a threshold of 100,000, and 91.8% and 98.5% by mcSUVR with a threshold of 1.5. Both *Z*-sum and mcSUVR showed high sensitivity and specificity. Most of the false-negative cases were visually equivocal [8/9 (88.9%) for the *Z*-sum and 7/8 (87.5%) for the mcSUVR].

**Fig. 7:**
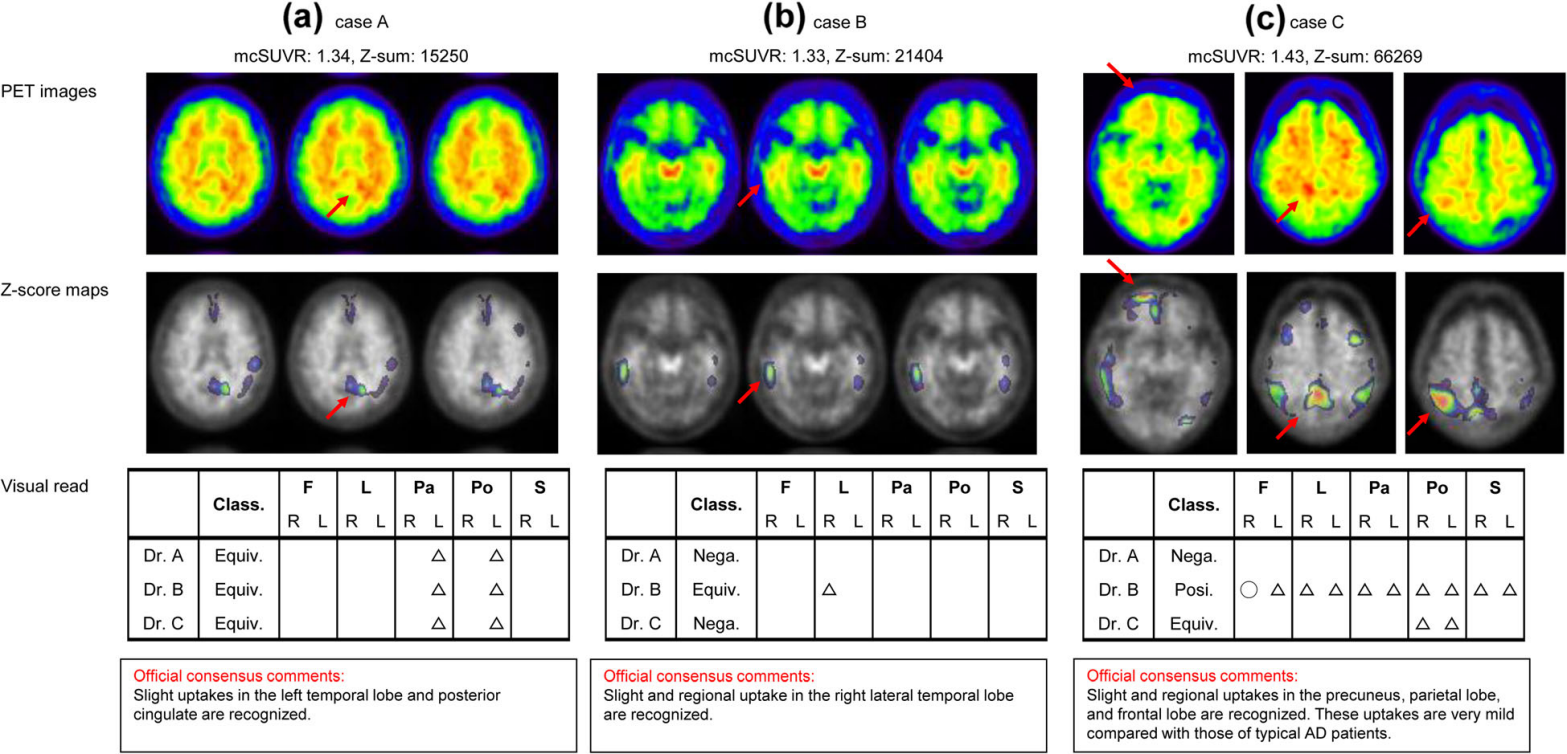
PET images and *Z*-score maps in MNI space, and the three physicians’ visual read and consensus comments for three representative cases, in which official consensus interpretation was “equivocal.” There are the cases in which three physicians' visual read matched (**a**) or did not match (**b** and **c**). The *Z*-score maps correctly delineated abnormal Aβ accumulation over the same regions as the visual read (red arrows). F, L, Pa, Po, and S are frontal lobe, lateral temporal lobe, lateral parietal lobe, posterior cingulate gyrus and precuneus, and striatum, respectively. Class., Nega., Posi., and Equiv. are classification, negative, positive, and equivocal, respectively. The circle and triangle represent the positive and equivocal region, as judged by the physicians

Three representative equivocal cases are shown in Fig. 7. The mcSUVR and *Z*-sum values of these equivocal cases were lower than their respective thresholds. As indicated by the red arrows in Fig. 7, the *Z*-score maps correctly delineated abnormal Aβ accumulation over the same regions as the visual interpretation even for the cases, in which the three physicians’ interpretation did not completely match.

## Discussion

We established voxel-based statistical analysis for amyloid PET. Once the normal database is created, this analysis can be fully automatically applied to amyloid PET without MRI. This voxel-based approach gives us an objective *Z*-score map for each amyloid PET image. Some previous studies showed that SPM software provided a brain surface map showing the difference between patients with AD and healthy control groups [15, 32]. Our proposed method, however, can provide a voxel-by-voxel *Z*-score map for each subject. This is more useful for physicians to complement their visual interpretations when reading amyloid PET images for individual subjects.

Both the quantitative values of mcSUVR and *Z*-sum showed high sensitivity and specificity. Most of the false-negative subjects were equivocal scans by visual interpretation. We believe that the *Z*-score map can effectively provide detection of Aβ accumulation in these cases. As shown in Fig. 7, for three representative equivocal scans, the *Z*-score maps correctly delineated the same regions as the individual and consensus visual reads. This was the case even in those whose mcSUVR was low. Although *Z*-score maps may not provide further benefit for obvious positive or negative cases, they may improve the Aβ detection sensitivity for cases with mild or limited regional Aβ accumulation. The *Z*-score maps also correctly delineated abnormal uptakes for the cases that three physicians’ interpretation did not match. Therefore, they might be useful in reducing inter-reader variability. Further study is warranted regarding the relationship between visual detection of abnormal or equivocal uptake and the maximum *Z*-score value within such areas. While the ROI-based quantitative approach can evaluate regional uptake to some extent, the proposed voxel-based approach can visualize voxel-wise Aβ accumulation in more detail. The proposed method may also provide closer monitoring of cerebral Aβ accumulation in follow up studies and treatment effect evaluations.

The NDB used in this study was derived from 18 Aβ-negative subjects including 2 AD patients, 6 MCI subjects, and 10 NC subjects. We confirmed that mcSUVR for each subject was smaller than 1.45 (Table 2). However, some MCI subjects showed somewhat higher maximum SUVR values compared with those of the NC subjects. These discrete high uptakes could affect the sensitivity of the *Z*-score mapping. If there are large datasets of NC subjects, it would be better to construct the NDB with only NC subjects.

In this study, voxel-based statistical analysis was successfully applied to a large dataset acquired with various PET scanners (Table 1). These results suggest that the proposed method would not be sensitive to the variability between PET scanners. As mentioned in the methods, before scanning the first subject, image resolutions of all PET scanners were harmonized by the Hoffman phantom test. This process might have effectively worked to minimize the difference between scanners. The NDB may be shareable if an appropriate reconstruction parameter is used so that the PET image resolution was 8 mm FWHM or better [25].

There are some limitations to this study. Firstly, the smoothing process and the parameters in creating the *Z*-score map have not been optimized. While the *Z*-score map was processed with a Gaussian smoothing filter of 8 mm FWHM, there is room for improvement in this point. Secondly, we used the PET-based spatial normalization method and the EPP-ROI template that we had proposed [23]. However, this approach may not completely eliminate contamination from white matter. When subjects have undergone MRI scans, MR-based spatial normalization and individual neocortical ROI would be less susceptible to contamination from the white matter. The white matter contamination might also affect the quantified value of cerebellar cortex in a different way. Thirdly, the *Z*-sum value largely depends on the number of calculation voxels. *Z*-sum was calculated by summing the voxel values within the EPP-ROI that were in the standard MNI space [23]. If we use a different ROI template and/or a different standard space [30], the appropriate threshold would likely be different. Lastly, we applied the proposed analysis only for ^11^C-PiB PET with the cerebellar cortex as the reference region. Our approach may be applicable to other amyloid or tau tracers [33, 34] but should be performed with a tracer-specific template, reference region, and database.

## Conclusions

We established voxel-based statistical analysis for amyloid PET and evaluated its feasibility using the J-ADNI datasets. The voxel-based analysis provides an objective *Z*-score map and a quantitative *Z*-sum value for each subject. These are helpful as an adjunct to visual interpretation, especially for cases with mild or limited regional Aβ accumulation. This approach could improve the Aβ detection sensitivity, reduce inter-reader variability, and allow for detailed monitoring of Aβ deposition.

## Data Availability

The clinical datasets analyzed in this article are available from the Japanese Alzheimer’s Disease Neuroimaging Initiative (J-ADNI) database deposited in the National Bioscience Database Center Human Database, Japan (Research ID: hum0043.v1, 2016).

## Abbreviations

AD: Alzheimer’s disease
EPP: Empirical PiB-prone
FWHM: Full width at half maximum
IBRI: Institute of Biomedical Research and Innovation
J-ADNI: Japanese Alzheimer’s disease neuroimaging initiative
MCI: Mild cognitive impairment
mcSUVR: Mean cortical standardized uptake value ratio
MNI: Montreal neurological institute
MRI: Magnetic resonance imaging
NC: Normal control
NDB: Normal database
OSEM: Ordered subset expectation maximization
PET: Positron emission tomography
ROC: Receiver operating characteristics
ROI: Region of interest
SD: Standard deviation
SPM: Statistical parametric mapping
SUVR: Standardized uptake value ratio
